# A Novel Integrated Transdermal Drug Delivery System with Micropump and Microneedle Made from Polymers

**DOI:** 10.3390/mi14010071

**Published:** 2022-12-27

**Authors:** Ajay Prabhakar Attiguppe, Dhiman Chatterjee, Amitava DasGupta

**Affiliations:** 1 Department of Mechanical Engineering, Indian Institute of Technology Madras, Chennai 600036, India; 2 Department of Electrical Engineering, Indian Institute of Technology Madras, Chennai 600036, India

**Keywords:** polymer valveless micropump, non-planar valveless micropump, polymer integrated drug delivery, transdermal drug delivery, polymer hollow microneedle, integrated drug delivery, microdosing system

## Abstract

Transdermal drug delivery (TDD), which enables targeted delivery with microdosing possibilities, has seen much progress in the past few years. This allows medical professionals to create bespoke treatment regimens and improve drug adherence through real-time monitoring. TDD also increases the effectiveness of the drugs in much smaller quantities. The use of polymers in the drug delivery field is on the rise owing to their low cost, scalability and ease of manufacture along with drug and bio-compatibility. In this work, we present the design, development and characterization of a polymer-based TDD platform fabricated using additive manufacturing technologies. The system consists of a polymer based micropump integrated with a drug reservoir fabricated by 3D printing and a polymer hollow microneedle array fabricated using photolithography. To the best of our knowledge, we present the fabrication and characterization of a 3D-printed piezoelectrically actuated non-planar valveless micropump and reservoir integrated with a polymer hollow microneedle array for the first time. The integrated system is capable of delivering water at a maximum flow rate of 1.03 mL/min and shows a maximum backpressure of 1.37 kPa while consuming only 400 mW. The system has the least number of moving parts. It can be easily fabricated using additive manufacturing technologies, and it is found to be suitable for drug delivery applications.

## 1. Introduction

In recent years, the requirements of modern therapeutics have placed a high impetus on research and the realization of systems and techniques for localized drug delivery. The benefits of such systems, such as improved drug stability and bioavailability, lower dosage, better patient compliance due to improved patient comfort, painless self-application, etc., have increased demand for such methods. Many localized delivery systems are possible such as liposome-encapsulated drugs, polymer-encapsulated drugs, micro-swimmers, nanoparticle-based drugs and transdermal drug delivery (TDD) [1,2].

TDD has many benefits, such as patient comfort due to it being almost pain-free, precise, targeted and localized drug delivery and dosing, reduced antimicrobial resistance and ease of use. These benefits make TDD very attractive from the end user perspective [3,4]. TDD allows for the creation of microdosing systems, which provide precise real-time control over the drug delivered, thereby allowing the creation of bespoke treatment regimens that can change from person to person and with time.

**Figure 1:**
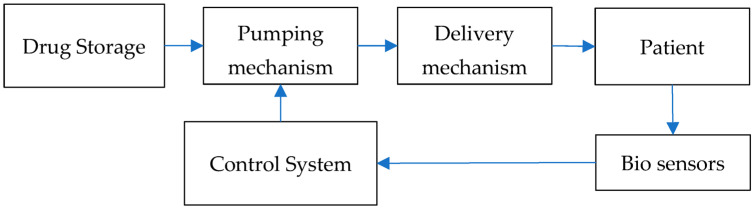
Schematic showing the major components in an integrated drug delivery (IDD) system.

An integrated drug delivery (IDD) system is presented in Figure 1. It consists of a storage/reservoir to store the drug, a pumping mechanism to drive the drug through the system, a delivery mechanism to deliver the drug into the system and a control circuitry to control the system. It can also consist of bio-sensors, which measure the changes in the body that the drug induces in real time. Data from the sensors can be used to alter the treatment protocols on demand through the control system. Designing such a system requires a careful choice of individual components and integration with each other. The system should be easily manufactured, be safe for human use and be cost effective in order to ensure large scale adoption. The delivery mechanism chosen should be capable of delivering the drug to at least 200 μm below the skin surface in order to reach the junction between the dermis and epidermis [5]. The pumping mechanism should be able to sustain drug flow against a backpressure of at least 400 Pa for delivering through the transdermal route [6]. The pump should work against the resistance of the system, the needles and the backpressure from the interstitial fluid. For sustained delivery, the maximum drug flowrate will be limited by the ability of skin to absorb the delivered drug. This flowrate has been shown to be 3–4 μL/min [7,8], and the micropump should be capable of sustaining this precisely. In this work we looked at an IDD system which uses a micropump as the pumping mechanism and microneedles as the delivery mechanism.

Microneedle based approaches have received much attention in the past decade due to tremendous developments seen in the field of microfabrication and researchers moving to use this in the field of drug delivery. Many drugs, in the form of microneedle patches, are either commercially available or are in various stages of clinical trials [9]. They can be used by patients themselves, reducing the load on the health systems. They help reduce the dosage of drugs and achieve desired bioavailability and therapeutic effectiveness at much lower doses than traditional drug delivery routes. Use of hollow microneedles helps overcome some of the hurdles faced by early generations of TDD such as limited molecular weight of the drugs that could be delivered, diffusion at a limited drug delivery rate and inability to deliver sensitive and macro molecules such as DNA, proteins and vaccines used in modern day treatments [10,11,12]. They allow real-time and precise microdosing, enabling creation of personalized treatments suited to individual patients instead of a one-size-fits-all approach. The hollow microneedle has been fabricated using different materials such as silicon, metals and polymers [13]. Polymer microneedles have the benefit of low cost, ease of fabrication, scalability and bio-compatibility. Among the many different methods reported for fabricating hollow polymer microneedles, the use of photolithography-based fabrication techniques allows faster, scalable and low-cost manufacturing. Hence, in this work, we use microneedles fabricated using a photolithographic process reported by us in an earlier work [14].

Another important component of the integrated system is the micropump. These devices have been widely researched and used in several fields. They are capable of transporting precise quantities of fluids over a wide range of backpressures while having a small footprint. Several different pumping mechanisms exist and many of them have found applications in drug delivery [15]. The chosen micropump should be able to operate using low power, be capable of delivering fluids against the backpressure provided by the skin, be drug and bio-compatible and, at the same time, have high reliability [16]. Micropumps can be classified as valved or valveless based on the rectification method used. Some of the earliest micropumps were passive valved micropumps where a flap acted as a valve to rectify the flow from the inlet to the outlet. They are capable of delivering liquids over a wide range of flowrates and backpressure, at the same time maintaining unidirectional flow. The biggest disadvantage of these pumps are the reliability and fabrication throughput, as the valves can become clogged or break during fabrication and handling. Valveless micropumps, on the other hand, can overcome this issue as they lack such delicate components. They use flow passages where flow in one direction is more than the other. The flow passages can be tesla valves, spiral tubes, Y-shaped tubes or nozzle and diffuser (ND) elements [17,18]. Owing to their ease of fabrication, ND-based valveless micropumps have received significant attention from researchers [19] and have been adopted in the present work. The flow rectification in ND elements occurs due to the pressure losses being different in the nozzle and diffuser directions. The rectification efficiency of an ND element can be defined as the ratio of pressure loss in the nozzle direction to that in the diffuser direction. This depends on the cone angle along with the entrance and exit conditions of the ND element [18,20]. The pumps can be further classified as in-plane [21], where the ND elements are on the same plane as the pump chamber, or can be non-planar [22], where the ND elements are not in the same plane as the chamber. Compared to planar micropumps, non-planar valveless micropumps offer the added advantage of a smaller footprint [22]. The earliest work on use of nozzle diffuser elements for flow rectification was reported by Stemme et al. [23], where they created a valveless micropump using conventional manufacturing techniques. Later, several researchers have reported ND-based micropumps having different ND element geometries [18].

Valveless micropumps have been produced using several materials and methods, with some of the earliest ones being made of metals and silicon [17]. Polymer micropumps provide an added benefit of drug and bio-compatibility, ease of manufacture and lower cost compared to other materials. Several researchers have developed micropumps using polymers having many different actuation methods [24]. The use of polymers opens up a wide variety of manufacturing techniques. Among them, additive manufacturing allows for creation of complex geometries easily and can be scaled up quickly. Though a lot of micropumps made of polymer have been reported, they tend to either lack the backpressure capacity required for drug delivery or have a large footprint, making them suitable for point-of-care systems rather than for transdermal drug delivery. Hence, in this work, we developed a polymer valveless micropump using a simple scalable method with a small footprint capable of meeting the requirements for drug delivery applications.

In the last decade, work has progressed in developing smart, integrated TDD systems involving a pumping mechanism, sensors and associated control circuitry. Some of these smart integrated systems are available in the market or are in the final stages of development. Some promising works have been made, with one of the earliest reports showing the development of an integrated insulin delivery system consisting of a microneedle and micropump [25]. This system used a silicon micropump with passive valves based on the Van Lintel design and silicon hollow microneedle array. After this, several researchers worked towards developing silicon-based systems owing to their ease of manufacture using established very large-scale integration (VLSI) fabrication processes. Debiotech^®^ is marketing an integrated delivery system with a small form factor using a silicon micropump and silicon microneedle array [26]. Though promising, these systems are inhibited by high cost of fabrication using sophisticated semiconductor processes and the specialized fabrication environments. More recently, focus has shifted towards developing polymer-based systems [9]. Use of polymers reduces cost, as well as improves bio-compatibility, and is easier to fabricate under much less demanding fabrication environments. Researchers are actively working on creating polymer micropumps [24] and polymer microneedles [14,27]. Recently, Mishra et al. [28] published a concept of an integrated system comprising an ion conductive polymer film (ICPF, Nafion 115) membrane-based micropump coupled with a polymeric hollow microneedle array. It is a low powered system capable of generating flowrates of up to 30 μL/min at 3–4 V input and 0.1–0.5 Hz of operational frequency. It is one of the few integrated systems published in literature in recent years that is mostly fabricated using polymers. The device, however, has the disadvantage of low backpressure performance and a limited flow rate range of the device. Moreover, the proposed system lacks an integrated reservoir and suffers from degradation of the membrane material over prolonged usage. Economidou et al. [29], demonstrated an integrated drug delivery platform using polymers. The work shows the integration of a 3D-printed microneedle array with a commercial polymer based micropump. The micropump shows an excellent microdosing accuracy with an error of just 7.9%. The major drawbacks of the system are the use of valved micropumps and large microneedles. The valved pumps can fail easily due to fatigue of the moving valves or due to the valve getting clogged. The use of large microneedles can cause increased pain and patient discomfort during application. Li et al. [30] reported an iontophoresis-based integrated drug delivery system. The system uses microporous solid microneedles to drive the drug into the transdermal layer of the skin using iontophoresis. The system is well suited for sustained drug release involving non-ionic drugs and is designed to have in-situ monitoring to enable closed loop control. The major drawback of such a system is the presence of an electrical field across the active drug component and the limited flowrate control. The system is specifically tailored for delivering insulin and lacks the ability to deliver drugs with suspended particulates or sensitive compounds. To the best of our knowledge, we report for the first time the development, fabrication and characterization of a non-planar piezoelectric valveless micropump integrated with a reservoir meant for storing the drug supply. Innovative pathways have been created using additive manufacturing for the fluid flow passage connecting the reservoir with the micropump, thereby making it a stand-alone system. Further, we present a novel integrated system suitable for TDD where this reservoir–micropump assembly can house a polymeric hollow microneedle array for drug delivery.

In Section 2, we discuss the design and fabrication of the components and the experimental setups used to characterize the system. In Section 3, we present the results of the characterization of the individual components as well as the integrated transdermal drug delivery system.

## 2. Materials and Methods

### 2.1. Design and Fabrication of Micropump, Microneedle and the Integrated System

As seen in the previous section, valveless micropumps are best suited for drug delivery applications. The pump should be able to sustain drug flow against a backpressure of at least 400 Pa while being able to sustain a flowrate of 3–4 μL/min precisely [6,7,8]. A pyramidal valveless micropump based on the design by Aggarwal et al. [31] has been shown to satisfy these conditions and, hence, was chosen as the starting point in this work.

This valveless micropump consists of an actuator to drive the flow, a chamber whose volume changes to create pressure and nozzle-diffuser (ND) elements connected to the chamber to rectify the flow. In the present work, we have integrated a reservoir to store the drug and a microneedle array to deliver the drug. Each of these components needs to be carefully selected in order to create a system that is capable of delivering the fluid (drug) at the desired flowrate and backpressure. It is crucial to ensure that the pump is airtight in order for it to create the pressure difference required to operate. Hence, the goal is to create an integrated system having a minimum number of parts requiring assembly, which is made of polymer and uses a scalable fabrication process. The system is designed to ensure minimal component assembly, which reduces the chances of pressure leaks and increases manufacturing output. Digital light processing (DLP)-based stereolithographic (SLA) fabrication was chosen due to its fast turnaround times, ability to produce precise parts with an internal cavity and channels with ease and low cost. The overall system has 3 components: the pump membrane, fluid passages and the microneedle array. The following sections present the design and creation of different components of the system.

#### 2.1.1. Pump Membrane

The pump membrane is the component that actuates and pushes on the liquid in the chamber, thereby increasing the pressure in the chamber. Among the many different actuation methods reported in the literature for use in micropumps, piezoelectric-based actuation is the most popular [17,24]. This actuation method shows fast response time, large actuation forces and simple design [31,32]. Use of readily available piezoelectric elements allows for simple integration with a membrane. PZT 5H-4E (piezo.com, Mide Technology, Woburn, MA, USA) was chosen as the piezoelectric material due to its large piezoelectric coefficient value. This piezoelectric element will be attached to a thin material. The composite structure acts as the membrane. On application of a voltage across its terminals, the piezoelectric material expands. As the element is constrained in the lateral direction, a stress is induced in the composite structure causing it to deflect out of plane, leading to a change in the volume of the chamber.

The deflection of the composite structure should be large in order to achieve a higher stroke volume. For this, the membrane should be thin enough to achieve a low stiffness. Stainless-steel is chosen as the membrane material as stainless-steel foils are readily available in various thicknesses and are bio-compatible. A membrane thickness of 30 μm is used for the foils in this work as they can be easily procured at low cost and are stiff enough compared to thinner SS foils, thereby ensuring that they do not deform easily during handling and PZT (lead zirconate titanate) mounting. A stiffer membrane also enables the pump to withstand higher backpressures.

**Figure 2:**
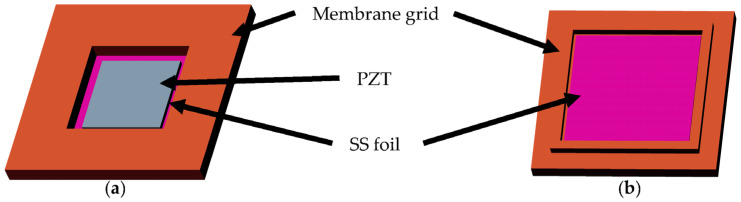
CAD model of the assembled composite membrane. (**a**) Top view. (**b**) Bottom view.

**Figure 3:**
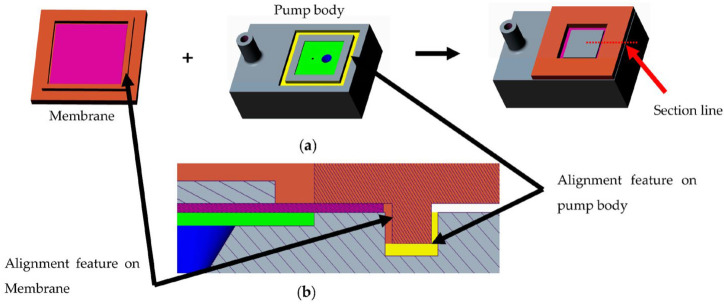
(**a**) CAD models of the individual components and the assembly. (**b**) Zoomed in view of the cross-section showing the alignment feature.

Previous work [31,32] has shown that the ratio of the lengths of PZT and membrane should be 0.85 in order to achieve the highest membrane deflection. Hence, a commercially available PZT plate of 8 mm × 8 mm is chosen to create the composite actuator. The PZT element is bonded onto the SS 304 foil to create a composite actuator, as shown in the computer-aided design (CAD) model in Figure 2. This composite structure is then attached to a 3D-printed grid. The grid is designed such that it allows precise alignment of the foil–PZT composite with it. This assembled structure also has alignment features to enable self-alignment of the membrane component with the pump chamber. The cross-sectional CAD model in Figure 3a shows how the composite membrane integrates with the fluid region. Figure 3b shows the zoomed view of the cross-section showing different regions of the pump along with the feature that aids self-alignment.

**Figure 4:**
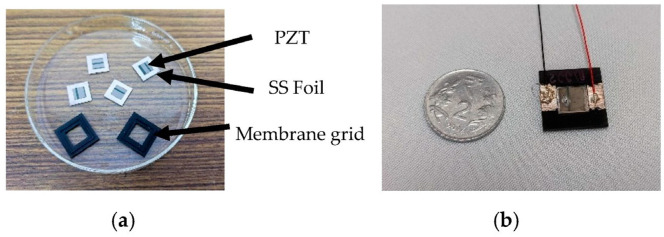
(**a**) PZT membrane composites and 3D printed membrane grids. (**b**) The assembled membrane component with the electrical connections.

Figure 4a shows the PZT-mounted composite membrane and the 3D-printed grids. Figure 4b shows the assembled membrane with the electrical contacts for actuating the membrane. The polymer grid is printed on a 3D printer (Figure 4 Standalone, 3D Systems, Rock Hill, SC, USA). Pro-BLK 10 bio-compatible photopolymer was chosen for its superior mechanical properties, low cost, bio-compatibility and its ability to create high resolution prints repeatably. An SS 304 stainless steel foil having an average thickness of 30 μm is cut into 13 mm × 13 mm pieces using a laser cutter. The PZT 5H-4E plates of 8 mm × 8 mm having a thickness of 127 μm are bonded to one side of the cleaned SS foil using a 2-part silver conductive epoxy from MG Chemicals (MG Chem 8331-14G, Ontario, Canada). This ensures that the membrane can be used as one of the terminals of the PZT. Silver paint (RS Pro Conductive Lacquer 186-3600, RS Pro, Corby, UK) is used to create an electrical connection from the membrane to the top surface of the plastic grid, to which a wire is bonded using the conductive epoxy. To bond the membrane PZT composite stack to the plastic grid in the seat provided on the polymer grid, a 2-part clear epoxy (Araldite Klear. Pidilite Industries Limited, Mumbai, India) is used.

#### 2.1.2. Fluid Region

The fluid region of the pump is composed of the pump chamber, the rectification elements and the drug reservoir. The inlet of the pump is connected to the reservoir, and the outlet is connected to the microneedle array.

The chamber planar dimensions are chosen to be 10 mm × 10 mm, based on the results from Aggarwal et al. [22], for their small footprint and their ability to pump against higher backpressure. The height of the pump chamber should be such that the liquid should not face significant resistance to flow between the inlet and outlet and at the same time should also avoid recirculation in the chamber. Since the chamber in the present design is created as a step on the top surface of the pump body, the achievable height depends on the chosen fabrication process. From trial and error, creating a 100 μm step height is found to be repeatable using the 3D printer (Figure 4 Standalone, 3D Systems) used in this work. When the composite membrane is assembled with the pump body, as described later, the step ensures that a chamber with a height equal to the step height is formed between the two components.

The out-of-plane valveless micropumps that use nozzle and diffuser (ND) elements to rectify the flow can be classified based on the cone angle of the ND element as a small angle (< 20°) or a large angle (> 20°) pump [20]. Both types of pumps have been researched extensively with the large angle ND element-based pumps receiving more attention, as they can be manufactured using standard CMOS silicon fabrication process. Additionally, the thickness of the pumps can be reduced with the use of large angle ND elements as the rectification efficiency reaches its maximum at smaller lengths compared to small angle elements [20,33]. A 70° cone angle is chosen for the large angle ND element. It has been shown that the static rectification efficiency is the highest when the cone angle is 70° for large angle ND elements [20]. Based on the direction of the net flow, the pumps can be either nozzle type, where the net flow is in the direction of the nozzle, or diffuser type, where the net flow is in the direction of the diffuser. A large angle pump can either be a nozzle or a diffuser pump, and the actual flow direction depends on the outlet cross section. Further, the length of the extension plays a lesser role in the rectification efficiency in the case of a nozzle pump than a diffuser pump [32]. Hence, in this work, a nozzle pump having equal cross-sectional areas at the inlet and outlet is chosen, thereby allowing us to vary the length of the extensions and giving design freedom for integrating the different components in a single structure.

The rectification efficiency of an ND element depends on the slenderness ratio, which is the ratio of the length of the ND element to the diameter of the throat. A minimum slenderness ratio of 1.5 is recommended for ensuring complete energy conversion from kinetic energy to pressure in a diffuser [34]. In the present work, a slenderness ratio of 2.8 is chosen for the ND element, as this is the minimum ratio above which rectification efficiency remains almost constant, as reported by Paul et al. [35]. The throat diameter is dependent on the fabrication method. An ND element having a throat diameter of 500 μm was found to be most optimal in terms of manufacturability by 3D printing.

The ND elements can be either pyramidal or conical. Traditionally, flat-walled ND elements have been preferred as they allow the length of the element to be small compared to a conical element when the desired direction of flow is along the diffuser. They are also easier to fabricate as planar elements using conventional manufacturing methods or as out of plane elements using standard silicon CMOS fabrication methods. In the present case, the desired flow is along the nozzle direction and the goal is to reduce the resistance to flow in this direction. Conical ND elements are more suited when the net flow direction is along the nozzle. This is because conical ND elements show lesser resistance to flow compared to the flat walled elements [36]. The use of additive manufacturing technologies also allows for creation of conical elements with ease.

**Figure 5:**
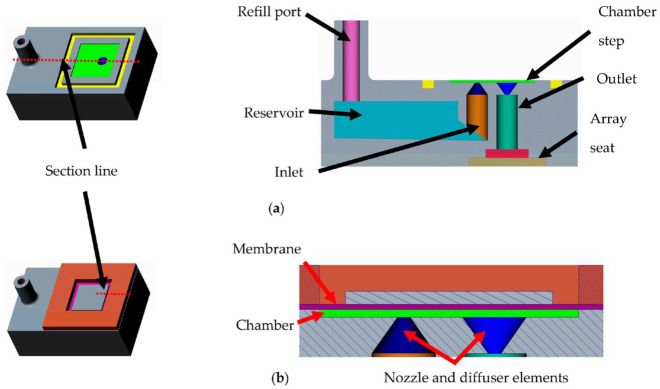
(**a**) CAD model of the pump body along with its cross section. (**b**) CAD model of the assembled pump along with its cross section.

Figure 5a shows the CAD model of the designed fluid region of the micropump. The reservoir is designed so as to be able to store 1 mL of the drug. It also shows a cross-section of the fluid region of the pump showing the reservoir and its location relative to the pump chamber. Figure 5b shows the CAD model of the assembled pump. The cross section in Figure 5b shows the chamber created once the composite membrane and the pump body are assembled. In order to ensure that the fluid does not flow out of the pump due to gravity, the reservoir is placed below the pump chamber. The inlet tube extends into the reservoir to within 1 mm of the reservoir floor. The reservoir floor is inclined by 2° towards the pump inlet in order to ensure that the entire drug in the reservoir is pumped. The other end of the reservoir opens through a refill port to atmosphere. This ensures that no vacuum is created as liquid is pumped out of the reservoir and also enables refilling. The outlet of the pump is connected to a needle plenum which opens out into a seat which will house the microneedle array. The height of the outlet should be kept close to that of the inlet in order to minimize the effect of siphoning.

**Figure 6:**
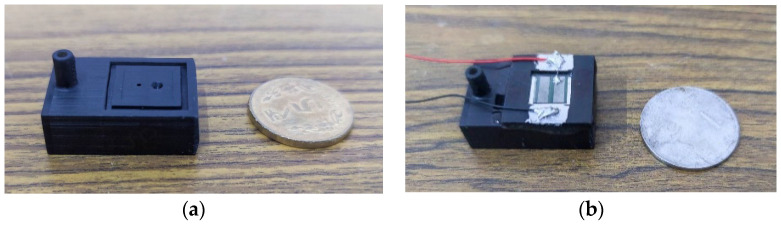
(**a**) The 3D printed fluid region of the system. (**b**) The fluid region assembled with the membrane component creating the complete pump.

The fluid region is printed using 3D systems Pro-BLK 10 photopolymer on the Figure 4 Standalone, which works on the DLP-based stereolithography. This allows the creation of the complete region as a monolithic component. In order to achieve successful prints, the model orientation on the print bed is tested for different configurations. Some design modifications such as a slanted inlet tube-end ensure a support-free repeatable print. Figure 6a shows the fabricated pump body. Next, the membrane component is assembled onto the fluid component. The membrane self-aligns with the chamber due to the design, as explained before. A 2-part clear epoxy (Araldite Klear) is used to join the two parts together. Figure 6b shows the completed assembly with the fluid and membrane components assembled. The entire pump has a small footprint of 30 mm × 20 mm with a height of 12 mm.

#### 2.1.3. Microneedle

A polymer microneedle fabrication process is reported by us in an earlier publication [14]. The force required for penetrating the needles should be minimal in order to reduce the pain felt during insertion [37]. A 5 × 5 array of needles having a triangular cross section with a circular lumen with a flat end and monolithically integrated to a SU-8 platform is fabricated. The spacing between the needles is 500 μm. The number of needles per array is reduced compared to the earlier work in order to ensure that the array size remains small while increasing the interspacing between the needles. A needle interspacing of 500 μm has been shown to be effective in penetrating skin [38]. The needles have a designed lumen diameter of 80 μm. The needle body is a triangular cross-section with a designed height of 178 μm. The resulting needles will have a lumen of 60 μm due to the diffraction of light in the lithographic process used for fabricating the needles. The complete array is 8.6 mm × 8.6 mm in size. As the needles should penetrate at least 200 μm into the skin and since skin is highly compliant, the needle height is designed to be 600 μm. The needles are designed to be monolithically integrated to a platform which holds the needles while providing mechanical support. A 400 μm thick platform is found to be mechanically stable while overcoming the patch warping that was faced during fabrication in our earlier work.

**Figure 7:**
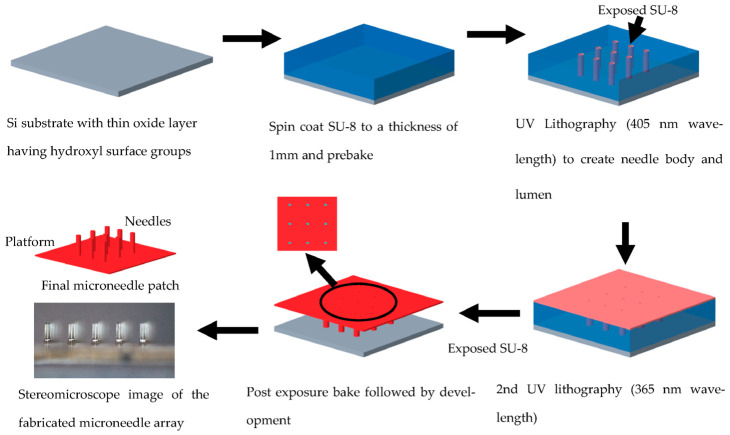
Schematic showing the microneedle fabrication process flow.

The needles are fabricated using SU-8 2150 (Kayaku Advanced Materials, Westborough, MA, USA). SU-8 is a negative photo polymer regularly used in the semiconductor industry. SU-8 2150 is a high viscosity variant of SU-8 designed for ultra-thick resist applications. Use of SU-8, which is bio-compatible, allows for adopting a simple fabrication process, as shown in Figure 7.

The fabrication process starts with a silicon substrate that provides mechanical support for the fabrication process. The silicon substrate is first cleaned in acetone and isopropyl alcohol (IPA) (Sigma Aldrich (Merck), Darmstadt, Germany). It is then cleaned further using a Piranha solution, which is a 3:1 mixture of H_2_SO_4_ (60 mL) and H_2_O_2_ (20 mL). Here, the silicon substrate surface is oxidized forming a thin layer of SiO_2_ with hydroxyl surface groups. This reduces adhesion of SU-8 onto the substrate surface [39] and allows for easier separation of the array once fabricated.

Next, a 1 mm thick layer of SU-8 2150 negative photo resist is spin coated onto the substrate. Here, the desired thickness of SU-8 is achieved by controlling the weight of the SU-8 on the substrate after the spin coating process, which was 1.4 g for a substrate of 3 cm × 4 cm. The spin coating only ensures that a layer of SU-8 covers the entire substrate to enable reflow of the resist under its own weight. Later, the resist reflows during pre-exposure bake, thereby forming a uniform layer of photoresist with very good planarity. The resist layer is subjected to a pre-exposure bake of 21 h carried out at 95 °C (for 11 h), with care taken to ensure a gradual ramp up and ramp down to minimize stresses and avoid wrinkle formation in the film.

The needle body, lumen and the platform are created using a 2-step lithographic process. In the first lithographic step, UV light of 405 nm wavelength (obtained by using a 4 mm thick non annealed acrylic filter) from a lithographic mask aligner (Karl Suss MA6/BA6, SUSS MicroTec, Garching, Germany) is passed through a photomask, which defines the body and lumen of the needle in the SU-8 film. The exposure energy used is 15.8 J/cm^2^. Next, without developing the exposed film, a dose controlled (80 mJ/cm^2^) second lithographic exposure with 365 nm wavelength is performed. Here, too, a photomask is used to align with the features produced in the first exposure. This step creates a 400 μm thick platform around the needles holding them together in an array and providing fluidic connection with the micropump.

The substrate is then subjected to a post exposure bake for 3 h at 90 °C (1 h) with gradual ramping of temperature. Then, the substrate is developed in an SU-8 developer with ultrasonication for 30 min. At the end of the development, the microneedle arrays separate from the substrate and are collected and dried. Finally, an array of polymer microneedle monolithically integrated with a platform is obtained.

#### 2.1.4. Integration

**Figure 8:**
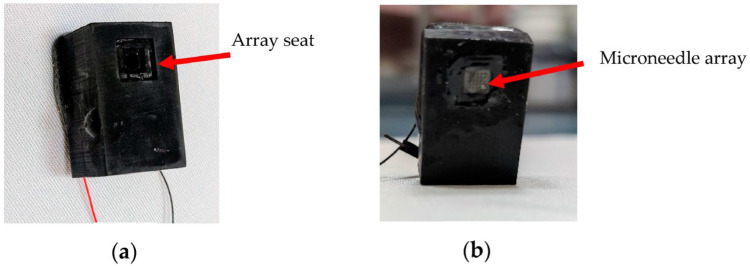
(**a**) The pump underside showing the needle seat. (**b**) The final system after assembling the micropump and the microneedle.

The microneedle array and the micropump with the integrated reservoir need to be assembled to create the final transdermal delivery platform. The design of the fluid region of the micropump is made such that the needle array can be fixed to the underside of the pump body. This allows the platform of the array to sit flush with the pump bottom allowing the needles to protrude out of the component. As mentioned earlier, this design reduces the siphon effect usually seen in valveless pumps with large chamber heights by reducing the height difference between the inlet and the outlet. A 2-part clear epoxy (Araldite Klear) is, again, used to bond the fabricated microneedle array to the needle seat (Figure 8a) on the underside of the assembled pump, as seen in Figure 8b. The fabricated components are subjected to mechanical and fluidic characterizations to analyze their performance. Table 1 shows the important geometrical and material parameters of the designed system. The following section discusses the various experiments conducted on the fabricated components.

### 2.2. Experimental Setups

#### 2.2.1. Microneedle

The fabricated microneedles are imaged using confocal microscopy (Olympus LEXT OLS4000, Olympus, Tokyo, Japan) and scanning electron microscopy, SEM, (SEC SNE4500M, sec, Gyeonggi-do, Korea). For SEM, the samples are gold coated on a tabletop sputter coater, and a scan voltage of 30 kV is used for imaging. The openings at both ends of the needles along with the height and outer needle dimensions are measured. The platform thickness is measured using a digital micrometer. Different mechanical tests were carried out on the fabricated microneedle array to assess the failure loads when subjected to bending and compressive loads. Fluid flow characterisation was also performed on the needles to measure the resistance the needles offer to water flow. The details of the experimental methodology adopted and the results obtained have been reported in our earlier work [14]. Based on that work, it was shown that the needles could withstand a force of 0.2 N in bending and a force of 0.7 N in axial loading. The needles offered minimal resistance to fluid flow of 0.2 Pa-min/μL for the flow of water.

**Figure 9:**
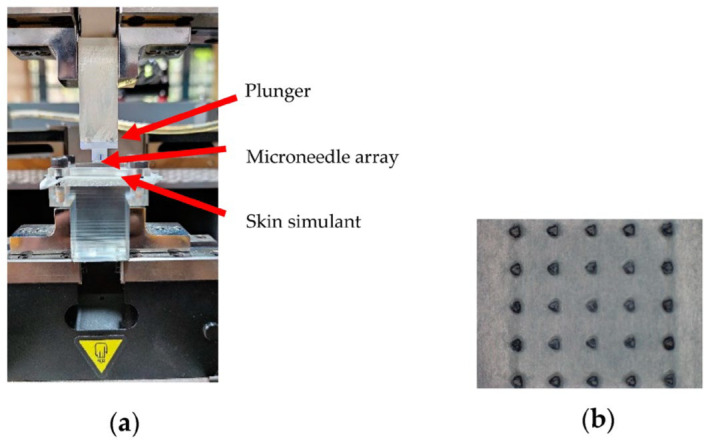
(**a**) The insertion test setup. (**b**) Dyed parafilm showing that the previous layer has been successfully penetrated.

Insertion tests are carried out using the fabricated microneedle arrays to determine if they could penetrate the skin. The needles are required to penetrate deep into the epidermis, which has a thickness between 100 and 200 μm [40,41]. As the mechanical properties of human skin vary from person to person and even at different regions of the body, a skin simulant was chosen for the tests. Parafilm-M, a commerically available laboratory film, has been shown to be an effective skin simulant for testing microneedle insertion capabilities [42,43] (Amcor, Zurich, Switzerland). Parafilm-M, having a thickness of 125 μm, is folded 8 times onto itself creating a testing film of approximately 1 mm thick. An expanded polyethylene layer is placed below the testing membrane and serves the purpose of simulating the muscle backing below the skin. A jig is designed and fabricated inhouse to hold the testing film while the needles attached to a plunger press the needles against the testing membrane. A micro tensile tester (Instron UTS 5948, Norwood, MA, USA) is used to carry out the testing, and the setup is shown in Figure 9a. The needles are plunged into the test film at a rate of 5 mm/min and, once a pre-determined force is reached, it is held pressed against the membrane at this force for 30 s. The needle array is then pulled away from the parafilm at a rate of 10 mm/min. Sudan black dye is applied on the parafilm after the test to help analyze if a layer has been penetrated by observing the dyed film under a microscope. A mark on one layer indicates that the previous layer has been penetrated, as seen in Figure 9b. This gives an approximate value for the penetration depth of the needle array.

#### 2.2.2. Micropump

The assembled micropump is subjected to fluid flow and mechanical characterizations to analyze its performance prior to attaching the microneedle array. This allows us to analyze the pumping characteristics of the pump and the effect of introducing the needle array on the pumping performance.

**Figure 10:**
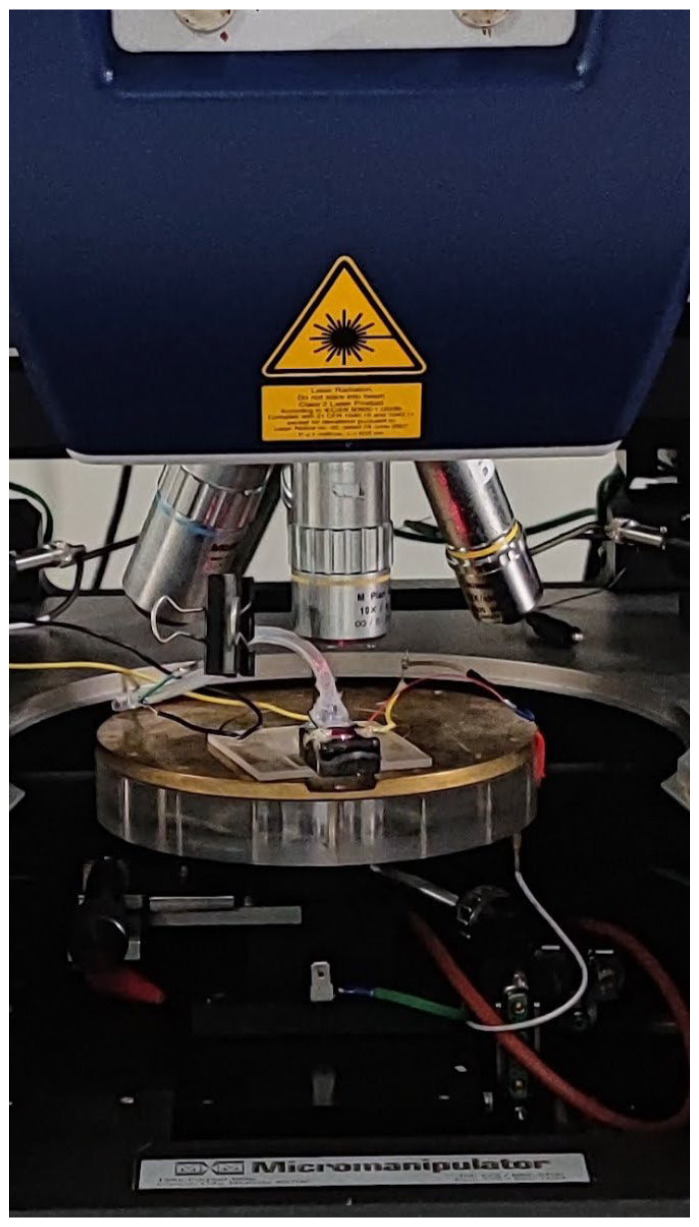
The measurement setup used to analyze the resonant frequency of the pump membrane.

The fundamental resonance frequency of the membrane with air and water as the working medium is measured. The displacement of the membrane and, hence, the performance of the pump is the maximum when the pump membrane is actuated at its fundamental frequency [35]. The fundamental frequency depends on the working medium. Hence, vibrational analysis was performed for the micropump with air and water as the working medium using a laser doppler vibrometer (Polytec MSA-500, Polytec Holding AG, Hörsching, Austria). Figure 10 shows the experimental setup. For water as the working medium, a silicone tube is connected to the reservoir inlet. Once the pump is primed with water, the tube is clipped. This ensures that the water does not flow out during measurements. The pump is excited with an 8 V periodic chirp signal, and a fast Fourier transform of the captured velocity data is used to identify the frequencies at which the pump membrane resonates.

**Figure 11:**
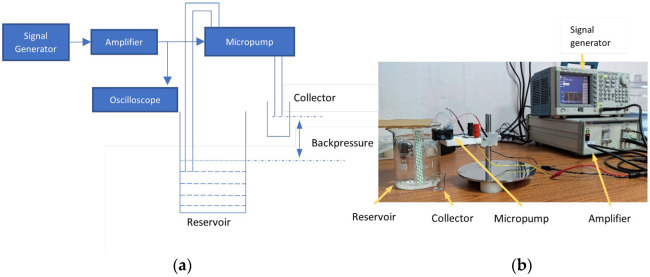
(**a**) Schematic of the experimental setup. (**b**) The experimental setup used to measure the performance of the micropump.

The assembled micropump is then subjected to a fluid flow characterization to analyze its performance with respect to the driving frequency, driving voltage and the backpressure against which the pump can operate. Figure 11a shows the schematic of the test setup, while Figure 11b shows the photograph of the actual experimental setup. Since the volume of the reservoir is limited, a silicone tube is connected to the inlet of the reservoir and is placed in a beaker. The water level in the beaker is kept constant, which ensures a constant backpressure for the duration of the testing.

Backpressure is measured as the difference in the level of water in the beaker and the outlet of the micropump. For testing the maximum backpressure of the pump, the level of water in the beaker is varied. The pump and the reservoir are filled with water and care is taken to avoid air in the flow path. A sinusoidal signal generated from a signal generator (AFG3151C, Tektronix, Beaverton, OR, USA) is amplified using a high voltage amplifier (2350, Tegam, Geneva, OH, USA) and applied across the terminals of the piezoelectric actuator. Current drawn is measured by connecting a micro ampere meter (15B+, Fluke, Everett, WA, USA) in series with the signal generator and the micropump. Water dispensed by the pump over a fixed period of operation is collected in a small glass beaker. The weight of the beaker is recorded using a precision weighing balance (AP225WD, Shimadzu, Kyoto, Japan) when empty and after water is collected. The difference in weight gives the mass of water pumped. From this, the net flowrate from the pump is calculated. The above procedure is carried out over a range of frequencies at 3 voltages of 50, 60 and 70 V_RMS_ to analyze the behavior of the pump. The frequency at which the flowrate is maximum is recorded. The backpressure performance of the pump is measured at different backpressures when the pump is operated at the frequency corresponding to the maximum flow rate at 3 voltages, as before.

Next, the microneedles are attached to the pump, as described earlier. The above mechanical and fluid flow characterizations are repeated on this integrated system, and the effect of adding the needle array on the system output is analyzed.

## 3. Results

### 3.1. Microneedle

Several needle arrays are produced using the process described in Section 2.1.3, and the process is found to be repeatable within experimental limits. Based on measurements made on five separate microneedle arrays, the needle lumen diameter is found to be 53.72 ± 10 μm, while the height of the triangular cross section is found to be 171.6 ± 15 μm. The height of the needles protruding from the platform is found to be 541.9 ± 50 μm with the platform itself measuring 450 ± 50 μm.

**Figure 12:**
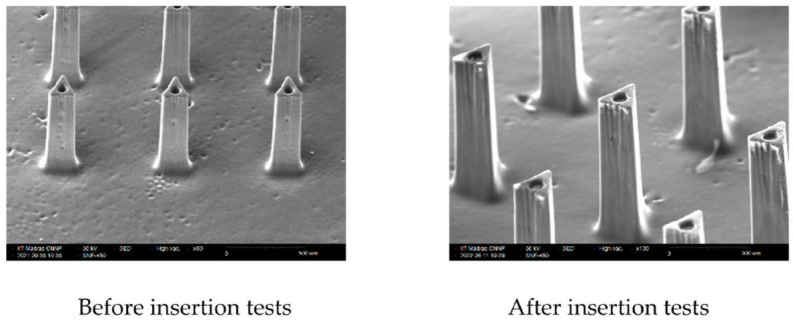
SEM images of the microneedle array taken before and after the insertion tests.

The skin penetration experiments carried out, as described in the previous section, showed that the needles are capable of penetrating 2 layers out of the 8 layers of parafilm used in the test. The penetration depth achieved corresponds to a thickness of approximately 250 μm, which satisfies the requirements for transdermal drug delivery. The average force over 3 trials required to achieve this is found to be 53.33 N for an array of 25 needles. When pressed against the parafilm layers, the needles fail when the force applied on the array was 90 N, which is well over the force required for insertion. It is to be noted that the failure load in compression presented in Section 2.2.1 is for the case where the needles are pressed against a rigid surface. It has been shown that the loads the needles can withstand significantly differ between the cases where the needle is pressed against a rigid material versus a soft material such as skin [44]. SEM images of the microneedle, as seen in Figure 12, taken before and after the skin penetration tests show no damage to the needles from repeated insertions.

### 3.2. Micropump

**Figure 13:**
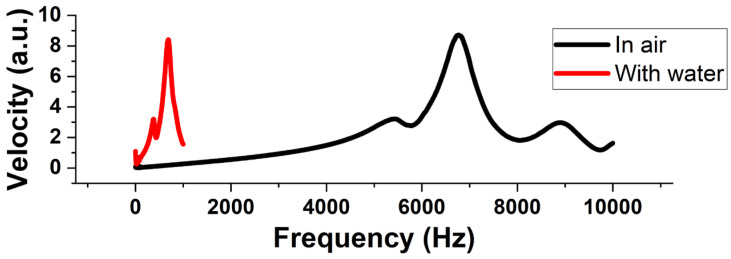
Frequency responses of the pump membrane when the working fluid is air and water.

From the vibration analysis performed on the micropump, the resonance frequency at first mode for the membrane when the pump is filled with air and water are obtained. Figure 13 shows the output obtained from the vibrometer showing the membrane velocity at different frequencies. The first peak is found to be at 6.7 kHz when air is the working medium, while the resonant frequency shifts to the left and is found to be at 689 Hz when water is the working medium. Addition of water in the pump chamber increases the effective mass of the oscillating substance and, thereby, causes a reduction in the resonant frequency. This phenomenon can be explained by considering an equivalent electrical network of the micropump presented by Bardell et al. [45]. When water is the working medium, the effective mass and, hence, the inductance of the membrane increases. This causes a reduction in the resonance frequency of the system.

**Figure 14:**
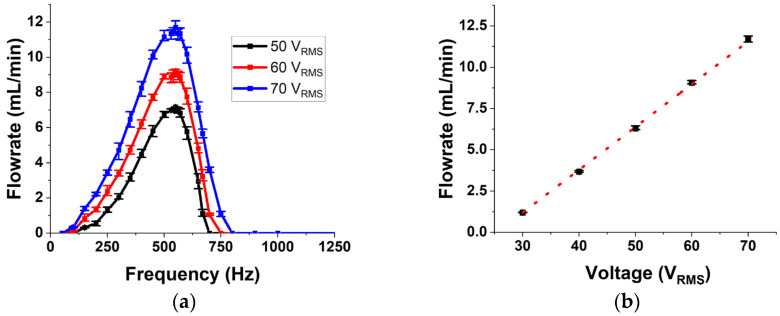
(**a**) The frequency response of flowrate of the pump. (**b**) Variation of maximum flowrate with applied voltage.

The results from the fluid flow experiments conducted on the micropump is presented in Figure 14. Flowrate is measured at operational frequencies between 0 and 2000 Hz to characterize its dependance on frequency. Figure 14a shows the experimental data as an average of 3 trials with the error bar showing the variation between the trials. The error bars indicate excellent consistency in the operation of the micropump. Initially the flowrate increases with an increase in frequency and then starts to decline. This behavior is in line with the flowrate vs. frequency characteristics reported in the literature for valveless micropumps [21,32,46]. Several reasons have been proposed over the years for this behavior. One of the reasons reported is that after a particular frequency, the fluid inertia dominates over the resistance, thereby increasing the overall flow impedance with frequency [32]. It has also been shown that the pressure applied by the membrane reduces after a particular frequency and that there is a phase lag between the applied input and the membrane deflection, which increases with frequency [21,46]. Another line of thought which looks at the performance of the nozzle and diffuser elements shows that these elements behave as low pass filters. After a cut-off frequency, the flow rate reduces drastically [47]. All these lead to a reduction in the net flowrate after a particular frequency, which will be called the peak performance frequency (PPF). The PPF for the fabricated pump is found to be at 550 Hz. This is found to be close to the membrane resonance frequency measured from the vibration analysis reported earlier. The difference in the membrane resonance and PPF can be due to the added inertia and stiffness of the complete flowpath compared to the membrane and chamber alone. This indicates that the peak performance of the pump will be at a frequency lower than the membrane resonance frequency.

Next, the behavior of the micropump at different operational voltages is presented in Figure 14b, which shows the variation of flowrate with voltage when the pump is operated at its PPF. This shows a linear relation between the applied voltage and the net flowrate produced by the pump. As the applied voltage increases, the membrane displacement increases linearly, thereby increasing the stroke volume. This in turn causes an increase in the flowrate.

**Figure 15:**
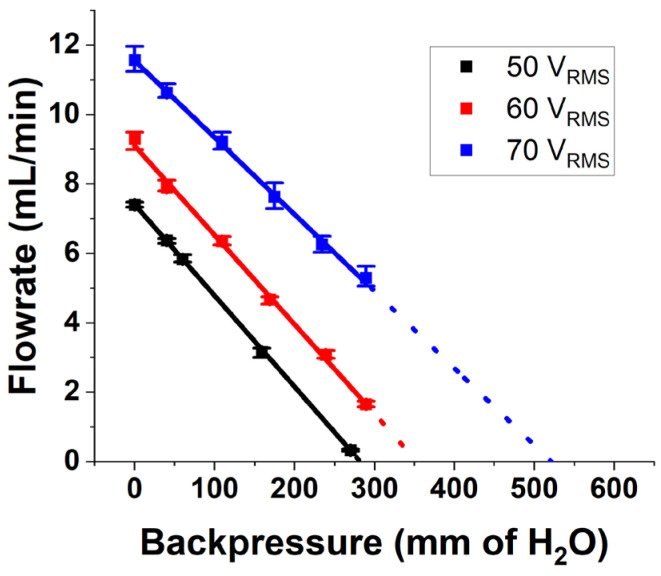
Variation of flow rate with backpressure when the pump is operated at 3 different voltages and at its PPF.

Another important parameter of the pump is its ability to pump against an adverse pressure at the outlet, also called the backpressure. Till now, the pump characteristics were measured when the pressure at the outlet of the pump was atmospheric pressure. In an application scenario, the micropump should deliver the drug against the pressure applied by the skin tissue. Figure 15 shows the flow rate from the pump at different backpressures when the pump is operated at its PPF and 3 operational voltages of 50, 60 and 70 V_RMS_. The graph shows a linear variation of flowrate with backpressure, which is a characteristic of valveless pumps reported in the literature [21,22,46]. It is seen that the maximum backpressure (backpressure at zero flow) obtained by extrapolating the experimental data when the pump is operated at 70 V_RMS_ and 550 Hz frequency is 5.15 kPa with a power draw of 400 mW. Hence, the pressure supplied by the pump is sufficient to overcome the tissue pressure of 400 Pa. A maximum flow rate (at zero backpressure) of 11.7 mL/min and the maximum backpressure (a zero flow) of 5.15 kPa at a low power of 400 mW are the important performance parameters of the fabricated pump when operated at 70 V_RMS_ and 550 Hz.

**Figure 16:**
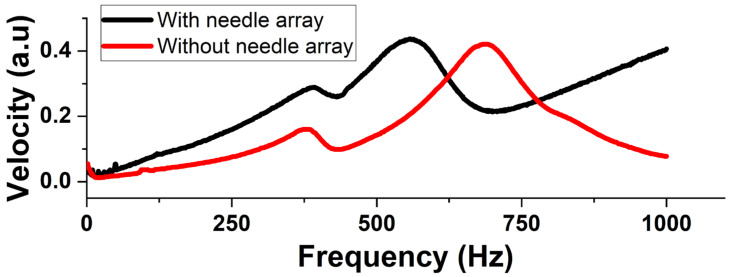
Comparison of membrane resonance with (in red) and without (in black) a microneedle array attached to the micropump.

Next, the microneedle array is attached to the underside of the pump. The above flow characterizations are repeated to analyze the performance of the integrated system. The goal is to understand the effect of adding an array of needles on the performance of the micropump and to analyze if the system is capable of meeting the requirements for drug delivery. The results of the vibration analysis conducted on the integrated system are shown in Figure 16. The velocity peak is found to be at 557.5 Hz. This reduction in the natural frequency can be attributed to a reduction in the effective stiffness of the membrane due to the added platform, which introduces a stiffness in parallel to the membrane.

**Figure 17:**
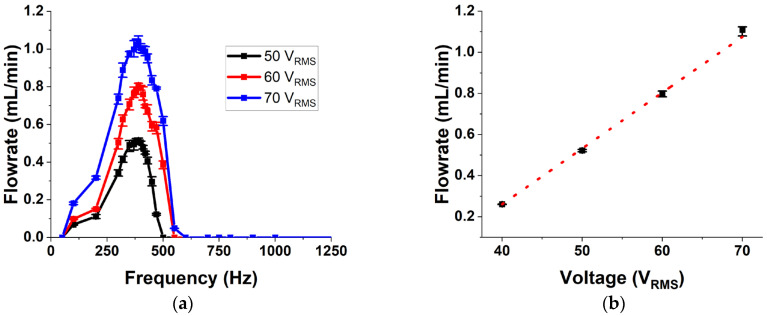
(**a**) Variation of flowrate with applied frequency. (**b**) Variation of flowrate with applied voltage.

Next, the performance of the system when the pump is integrated with the microneedle array is analyzed. First, the flowrate is measured at operational frequencies between 0 and 2000 Hz to characterize its dependance on frequency. Figure 17a shows the experimental data as an average of 3 trials with the error bar showing the variation between the trials. The error bars indicate excellent consistency in the operation of the integrated system. The behavior is very similar to that of the standalone pump. Howerer, the PPF shifts to the left, and the maximum flowrate is also less. This is in line with our prediction that the addition of the microneedle array increases the flow impedance of the system, thereby reducing its natural frequency as well as the net flowrate. The maximum flowrate is found to be 1.03 mL/min at 390 Hz and 70 V_RMS_ input signal and zero backpressure. The system showed a linear dependence on the the applied voltage, which is same as that of the standalone pump. As seen in Figure 17b, the addition of the microneedle array does not affect the linear dependance of the membrane deflection on the applied voltage and, hence, the trend remains linear.

**Figure 18:**
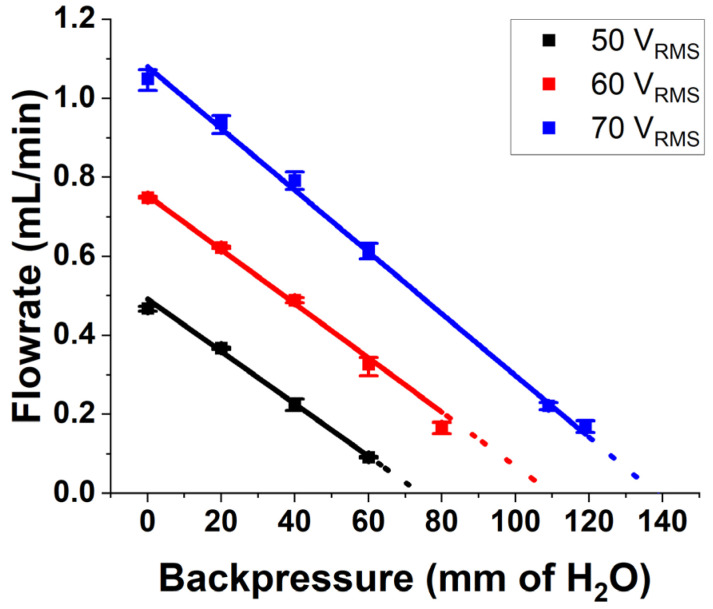
Variation of flowrate with backpressure when the microneedle array is attached to the pump.

One of the major parameters to be satisfied by the integrated system is to be able to deliver against a backpressure of at least 400 Pa. Figure 18 shows the flowrate from the system at different backpressures when it is operated at its PPF and 3 operational voltages of 50, 60 and 70 VRMS. The graph shows a linear variation of flowrate with backpressure, indicating that the addition of the microneedle array only increases the flow impedance and does not cause a change in the pressure characteristics of the micropump. It is seen that the maximum backpressure (backpressure at zero flow) obtained by extrapolating the experimental data when the pump is operated at 70 VRMS and 390 Hz frequency is 1.37 kPa. For a backpressure of 400 Pa and the same operating conditions, the system is capable of delivering fluid at 0.8 mL/min, which is more than the rate at which skin can absorb the drug, which is reported to be 3–4 μL/min [7,8].

## 4. Conclusions

In the previous sections we have presented the design, fabrication and characterization a of proof-of-concept integrated drug delivery system made of polymer using additive manufacturing technologies. For the first time in the literature, we report the fabrication and characterization of a non-planar valveless micropump made of polymer through additive manufacturing technologies. The fabricated pump shows a maximum flowrate of 11.7 mL/min and a maximum backpressure of 5.15 kPa with a very small power consumption of 400 mW, making it suitable for use in portable as well as wearable systems such as for transdermal drug delivery. As seen in Table 2, the developed integrated system is capable of satisfying the requirements for use in transdermal drug delivery. The system has the least number of components produced with a simple fabrication method. The proposed integrated design has the least number of components with a simple fabrication method. The integrated system shows a maximum flowrate of 1.03 mL/min and a maximum backpressure of 1.37 kPa. These parameters are much higher than the backpressure of 400 Pa and flowrates of 3–4 μL/min required for delivering drugs through the transdermal route. Future work would involve introducing an applicator to assist in generating the forces required to penetrate the skin as well as introducing sharp tipped microneedles to further reduce the penetration forces.

## Figures and Tables

**Table 1:** Geometric and material parameters of different components of the designed TDD system.

Parameter	Value
Membrane size	10 mm × 10 mm
Membrane thickness	30 μm
Membrane material	SS 304
PZT size	8 mm × 8 mm
PZT thickness	127 μm
PZT material	PZT-5H4E
Chamber size	10 mm × 10 mm
Chamber height	100 μm
ND element throat diameter	500 μm
ND element height	1.4 mm
Distance between ND element axes	3.5 mm
Material of the pump body	Pro-BLK 10
Microneedle height	600 μm
Microneedle lumen diameter	80 μm
Height of the triangular microneedle cross section	178 μm
Spacing between needles	500 μm
Microneedle material	SU-8 2150

**Table 2:** Important specifications of the developed IDD System.

Parameter	Requirement	Achieved
Microneedle insertion depth	>200 μm	250 μm
Microneedle factor of safety	>1	1.8
Micropump backpressure		5.15 kPa
Micropump flowrate		11.7 mL/min
Micropump power consumption		400 mW
IDD flowrate at 400 Pa backpressure	3–4 μL/min	0.8 mL/min
IDD max backpressure	>400 Pa	1.37 kPa

## Data Availability

All data generated in the study are available within the results section.
